# Polycyclic aromatic hydrocarbons in US and Swedish smokeless tobacco products

**DOI:** 10.1186/1752-153X-7-151

**Published:** 2013-09-08

**Authors:** Kevin G McAdam, Arif Faizi, Harriet Kimpton, Andrew Porter, Brad Rodu

**Affiliations:** 1 British American Tobacco, Group Research and Development, Regents Park Road, Southampton SO15 8TL, United Kingdom; 2 3810 St. Antoine W., Montreal, QC H4C 1B4, Canada; 3 University of Louisville, Clinical Translational Research Building, 505 South Hancock Street, Louisville, KY 40202, USA

**Keywords:** Smokeless tobacco, Snus, Polycyclic aromatic hydrocarbons

## Abstract

**Background:**

Debate about the health implications of using smokeless tobacco products (STPs) has prompted considerable interest in characterising their levels of toxic and carcinogenic components. In the present study seventy smokeless tobacco products from the US and Sweden, categorized as chewing tobacco, dry and moist snuff, hard and soft pellets, plug, and loose and portion snus, were analysed for twenty one polycyclic aromatic hydrocarbons (PAHs). The tested brands represented 80-90% of the 2008 market share for the major STP categories in these two countries.

**Results:**

There were significant differences in the total and individual PAH concentrations in the different styles of product. Substantially higher levels of total PAHs (10–60 fold) were found in moist and dry snuff and soft pellets than in the other smokeless tobacco styles. The individual PAH concentrations followed the same patterns as total PAHs except for naphthalene, for which the highest concentrations were found in snus and moist snuff. Good correlations were obtained between benzo[*a*]pyrene (B[*a*]P) and all the other PAHs except naphthalene, 1-methylnaphthalene and 2-methylnaphthalene, providing evidence for the first time that it can be used as a good marker for PAHs in STPs. Results were generally in good agreement with two previous studies of PAHs in STPs, except for naphthalene for which significantly lower concentrations were found than previously reported. Analysis of the ratios of different PAHs confirmed that the use of fire-cured tobaccos in the snuffs and soft pellet were the major source of PAHs in these product styles, and provided, for the first time, some indications as to the source of PAHs in the other STP styles, including petrogenic and other combustion sources.

**Conclusions:**

This study confirms the presence of PAHs in STPs, and identifies substantial differences between the levels in different STP categories. Since previous studies of naphthalene concentrations in STPs differed so markedly from those found in this study, it is recommended that further work on PAH determination is undertaken to investigate the source of this discrepancy.

## Background

There has been considerable interest in recent years in the chemical composition of smokeless tobacco products (STPs), primarily related to health concerns associated with their use. The International Agency for Research in Cancer (IARC) has classified smokeless tobacco as carcinogenic to humans (Group 1). IARC Monograph 89 [1] summarised the identification of 28 carcinogens in STPs including a number of tobacco specific nitrosamines, benzo[*a*]pyrene (B[*a*]P), metals, volatile nitrosamines and aflatoxins. More recently the World Health Organisation (WHO) Study Group on Tobacco Product Regulation (TobReg) recommended limits on the levels of several of these toxicants, including B[*a*]P, in STPs [2]. In 2012 the US Food and Drug Administration (FDA) established a list of Harmful and Potentially Harmful Constituents (HPHC) in tobacco products and tobacco smoke [3]. The list contains 93 compounds, of which 14 are polycyclic aromatic hydrocarbons (PAHs). For most of these compounds there are no standard analytical methodologies, and the FDA currently requires manufacturers to report levels of 9 HPHC in STPs [4], including one PAH, B[*a*]P.

PAHs, including B[*a*]P, are a group of chemicals that are formed during the incomplete burning of organic material such as coal, oil, gas, wood, tobacco and charbroiled meat. PAHs generally occur as complex mixtures (for example, as part of combustion products such as soot), not as single compounds. In tobacco smoke, for example, more than 575 different PAHs have been identified [5]. PAHs do not occur naturally in plant material, and where present their occurrence is due to contamination from combustion exhausts [6]. For tobacco, in particular, the curing process can introduce PAHs to the leaf if the tobacco is exposed to exhaust gases from heat sources that rely on burning wood or other organic fuels [7]. Fire cured tobaccos, whose production involves direct contact of the leaf with wood-smoke, contain particularly high concentrations of PAHs [8].

B[*a*]P is the only PAH in tobacco and tobacco smoke that is classified as a Group 1 carcinogen by IARC [1], and there are well established methods for its determination in tobacco and smoke, so its concentration is often used as a surrogate for the overall smoke concentration of PAHs. Likewise, B[*a*]P has been used as a surrogate for the presence of PAHs in tobacco leaf, although its utility as a PAH marker with STPs has yet to be validated. Its presence in smokeless tobaccos has been a focus of concern in the public health community as a result of several surveys [8-13].

However, even though there have been 86 PAHs reported to be present in tobacco [5], there is little quantitative information available on the levels of PAHs other than B[*a*]P in STPs. The most comprehensive study published to date is that of Stepanov et al. [12] who quantified the levels of 23 PAHs in US moist snuff and portion snus.

Given the focus on B[*a*]P in regulatory environments, and the lack of quantitative information on PAHs in STPs other than moist snuff and US snus, there is clearly a need for more information about PAH levels in contemporary STPs. The current study focused on establishing the PAH profiles of a greater range of smokeless tobacco styles than currently available in the literature.

### STP styles and brands tested

#### STP styles

The STPs analysed in the current work comprised eight different product styles: American dry snuff, moist snuff, chewing tobacco, plug, hard pellet, soft pellet and Swedish loose and portion snus. The following descriptions of the different types of product were derived from a standard glossary for smokeless products recently published by the CORESTA Smokeless Tobacco Sub-Group [14]:

##### Dry Snuff (DS)

US DS has the appearance of a fine brown powder with a moisture content of about 10% or less. DS usually contains a significant proportion of fire-cured tobacco. As used in the US, DS is placed between the cheek and the gum.

##### Moist Snuff (MS)

Also known as dipping tobacco, US MS is available as fine cut or medium/long cut tobacco particles, and contains air-cured and fire-cured tobaccos that are blended and fermented. The final moisture content is typically 50-60%. The products are usually placed between lower lip and gum and require expectoration during use; they are available both loose and in individually portioned sachets.

##### Chewing Tobacco (CT)

Loose leaf CT that is used in North America typically consists of loosely packed cut, or strips of, stem-free tobacco leaf which is cased with sugars and flavourings. The final moisture content is usually higher than 15%.

##### Plug

A form of CT traditionally used in North America. The product typically contains flaked tobacco leaves to which other ingredients may be added. The final moisture content is typically higher than 15%. The product has the appearance of a compressed brick wrapped inside a natural tobacco leaf.

##### Tobacco Pellets (HP, SP)

Two forms of tobacco pellets were examined: a hard pellet (HP) containing fine ground tobacco and inorganic materials, with a moisture content of around 5-10%, which is consumed by allowing it to dissolve in the mouth. This type of product is also termed dissolvable tobacco. There was also a soft pellet (SP) product consisting of a small cylinder of flavoured leaf tobaccos at a moisture of about 20%. This is kept between cheek and gum until the flavour has dissipated. The SP is also described as CT bits.

##### Snus (L Snus, P Snus)

Snus are smokeless tobacco products traditionally used in Scandinavia and are available in loose (L Snus) or portion (P Snus) styles. They are manufactured from heat treated tobacco that is processed into fine particles. The final moisture content is typically higher than 40%. Semi-dry products (less than 40% moisture) are also available. The products are usually placed between the upper lip and gum, and do not require expectoration during use. Swedish Match introduced the Gothiatek® manufacturing quality standards which, in part, sets upper limits on the concentrations of several toxicants including B[*a*]P [13].

#### Brands tested in the survey

The survey was conducted by sampling 70 STPs from the US and Sweden. Details of the markets in the US and Sweden were obtained in 2008 and the products for investigation were selected to cover all the major manufacturers and to provide information on products representing approximately 90% market share of the major STP categories (MS, CT and snus) for these two markets (Additional file 1: Tables S1 and S2). For DS the products chosen represented >42% market share. The HP and SP products are essentially single-manufacturer products, and therefore market share data was not relevant to these categories. For this survey, commonly available products were chosen from these pellet products. US market share data was obtained from a commercially available report [15]; Swedish product market shares were obtained using market monitoring by British American Tobacco (BAT) staff. One or more members (usually unflavoured, although some flavoured exemplars were chosen) of brand families were selected for analysis. It should be noted that the market shares listed in Additional file 1: Tables S1 and S2 include all flavour variants of the same brand family.

In total the survey comprised:

● 32 Swedish products: 10 L snus and 22 P snus (Additional file 1: Table S1). These were sourced from Swedish retail websites in 2008, imported into the UK and kept frozen at -20°C until tested. The products represented 7 different manufacturers.

● 38 US products: 13 CTs, 5 DSs, 2 HP products, 1 SP product, 16 MSs and 1 plug product (Additional file 1: Table S2). These were purchased from shops in North Carolina, US in 2008. They were imported and kept frozen, as above. The products represented 9 different manufacturers.

In all cases one sample (tin) of each brand was used for analysis.

### Polycyclic aromatic hydrocarbons (PAHs)

Twenty one PAHs were measured in this survey. These were: naphthalene (NAP), 1-methylnaphthalene (1-MN), 2-methylnaphthalene (2-MN), acenaphthylene (ANY), acenaphthene (ANE), fluorene (FLN), phenanthrene (PHEN), anthracene (ANTH), fluoranthene (FLNT), pyrene (PYR), benzo[*a*]anthracene (B[*a*]A), chrysene (CHR), benzo[*b*]fluoranthene (B[*b*]F), benzo[*k*]fluoranthene (B[*k*]F), benzo[*j*]fluoranthene (B[*j*]F), benzo[*e*]pyrene (B[*e*]P), benzo[*a*]pyrene (B[*a*]P), perylene (PER), dibenz[*a*,*h*]anthracene (DB[*ah*]A), indeno[*1,2,3-cd*]pyrene (I[*cd*]P), and benzo[*ghi*]perylene (B[*ghi*]P). The list of PAHs measured and their structures are given in Table 1.

**Table 1:** **Polycyclic Aromatic Hydrocarbons (PAHs) measured and Limits of Detection (LOD) and Quantification (LOQ)**^**1**^

**Chemical name**	**LOD (ng/g)**	**LOQ (ng/g)**	**Structure**	**Chemical name**	**LOD (ng/g)**	**LOQ (ng/g)**	**Structure**
Naphthalene	0.063	0.210		1-methylnaphthalene	0.038	0.126	
2-methylnaphthalene	0.028	0.095		Acenaphthylene	0.028	0.095	
Acenaphthene	0.060	0.200		Fluorene	0.029	0.098	
Phenanthrene	0.022	0.075		Anthracene	0.029	0.096	
Fluoranthene	0.025	0.082		Pyrene	0.059	0.197	
Benzo[*a*]anthracene	0.046	0.152		Chrysene	0.029	0.097	
Benzo[*b*]fluoranthene	0.106	0.352		Benzo[*k*]fluoranthene	0.074	0.247	
Benzo[*j*]fluoranthene	0.141	0.471		Benzo[*e*]pyrene	0.043	0.145	
Benzo[*a*]pyrene	0.066	0.221		Perylene	0.071	0.237	
Dibenz[*a*,*h*]anthracene	0.077	0.258		Indeno[*1*,*2*,*3*-*cd*]pyrene	0.063	0.211	
Benzo[*ghi*]perylene	0.063	0.211					

^1^LOD and LOQ specified by Labstat for PAH in samples of processed tobacco (Method TWT-335) on a wet weight basis.

## Methods

### Moisture contents

Moistures of the STPs were determined by Labstat International (Labstat International ULC, 262 Manitou Drive, Kitchener, ON, Canada N2C 1 L3) using a gravimetric oven moisture method [16].

### PAHs

**Figure 1:**
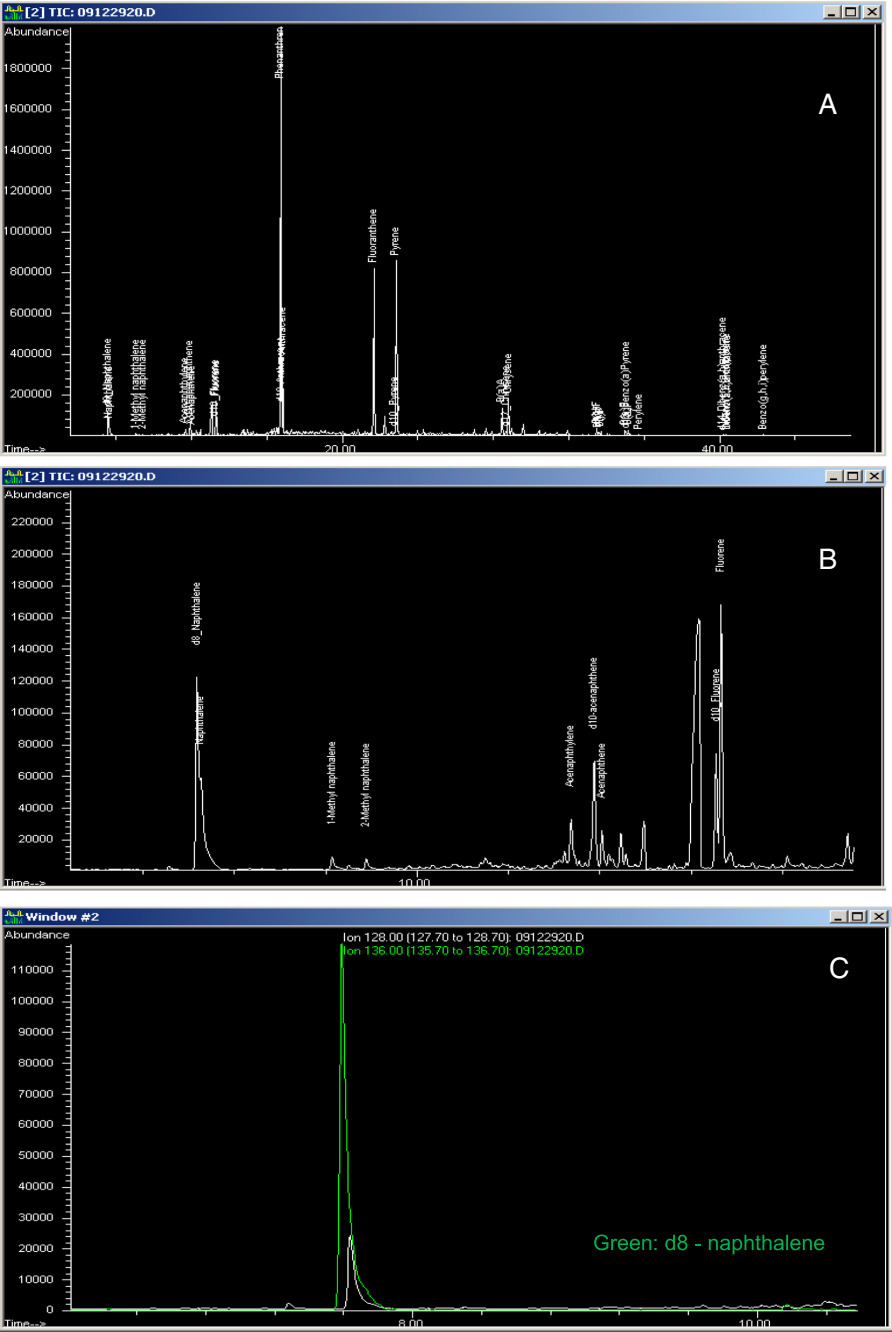
**Examples of chromatograms. A**: Total ion chromatogram for Timberwolf Straight Long Cut. **B**: Same chromatogram on an expanded scale. **C**: Expanded chromatograms for m/z 128 (naphthalene) and m/z 136 (d8-naphthalene).

The twenty one PAHs were determined at Labstat International (Method TWT–335) by extraction of the STPs using base saponification and partitioning followed by gas chromatography/mass spectroscopy (GC/MS) analysis [17]. In summary, 2 g samples were taken from a single container of each STP. A mixture of internal standards (8 deuterated PAHs – comprising deuterated analogues of naphthalene, phenanthrene, anthracene, benzo[*a*]anthracene, benzo[*a*]fluoranthene, benzo[*a*]pyrene, dibenz[*a,h*]anthracene, and benzo[*g,h,i*]perylene), were added to the STP sample pre-extraction and allowed to equilibrate before refluxing for 2 hrs with 60 mL of reagent alcohol and 4.5 mL of 50% potassium hydroxide. The mixture was partitioned into iso-octane, the iso-octane extract evaporated using a rotary evaporator and the concentrated sample passed through a 3 mL amino (200 mg) plus silica gel (750 mg) (SPE) cartridge. The retained PAHs were eluted with 13 mL hexane, and the eluate was evaporated to 2 mL with a TurboVap. Analyses were performed by GC/MS in selected-ion-monitoring mode, using a 30 m ZB-50 (0.25 mm × 0.25 μm) column with injection volumes of 1-3 μL. An injector temperature of 300°C, an interface temperature of 280°C and source temperature of 230°C were used in combination with a gc temperature program starting at 70°C for 1 second and ramping at 10.5°C/min to a final oven temperature of 300°C. Quantification ions and recoveries were as follows: naphthalene (quantification ion (qi): 128, recovery (r):104%), phenanthrene (qi: 178, r: 90.5%), anthracene (qi: 178, r: 90.1%), benzo[*a*]anthracene (qi: 228, r: 98.2%), benzo[*a*]fluoranthene (qi: 252, r: 85.5%), benzo[*a*]pyrene (qi: 252, r: 104%), dibenz[*a,h*]anthracene (qi: 278, r: 95.9%), and benzo[*g,h,i*]perylene (qi: 276, r: 98.2%); relative standard deviations were under 11%. The limits of detection and quantification for the 21 PAHs are given in Table 1, and representative chromatograms are shown in Figure 1. Chromatogram A is the total ion chromatogram for Timberwolf Straight Long Cut. B shows the same chromatogram but on an expanded scale. C shows the expanded chromatograms for m/z 128 (NAP) and m/z 136 (d8-NAP).

### Statistical tests

One way analyses of variance (ANOVAs) were performed using the Minitab (Version 16) statistical package (Minitab Inc, State College, Pennsylvania, USA) to indicate differences between PAH contents of STPs. Results were analysed using the Tukey method. Tests for statistical significance were set at the 95% confidence level.

## Results

STPs have a wide range of moisture contents largely according to the different product styles to which they belong. This has prompted discussion [18] as to whether the relative concentrations of toxicants in STPs should be compared on a wet weight (WWB) or dry weight (DWB) basis. Since the user is exposed to PAHs in the moist product it can be argued that it is more relevant to compare WWB concentrations. However DWB concentrations account for variability in moisture and permit comparisons across different STP categories. This latter approach is used in regulatory and industry proposals for limiting concentrations of toxicants such as B[*a*]P in STPs. Given the value in both forms of measurement, both WWB (measured) and DWB (calculated) concentrations of the PAHs will be discussed in this study.

In reporting and discussing the results of this study we first examine the moisture contents of the study STPs before examining variations in PAHs across product styles. Naphthalene, which appears to have a different distribution than the other PAHs, is discussed in a separate section.

### Moisture contents

**Figure 2:**
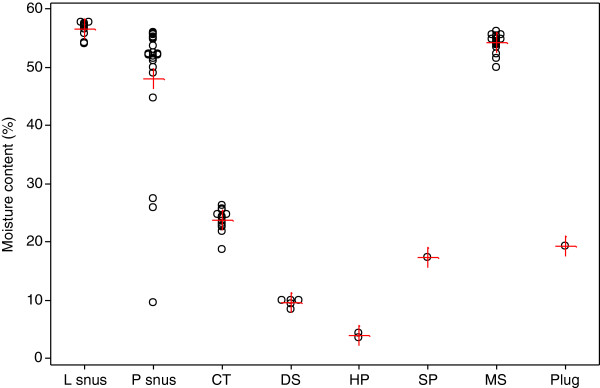
**Ranges of moisture (individual and mean values,%) in the STP brands by product style.** Individual values are represented by open black circles, means by red crosses.

Moisture contents of the STPs are shown in Additional file 2: Table S3 for the Swedish and American STPs respectively. The mean values and ranges of moisture contents obtained in this study for each style are summarised in Table 2 and illustrated in Figure 2.

**Table 2:** Averages and ranges of moistures (%) and total PAH concentrations (ng/g DWB) by product style

**Style**	**Number of brands**	**Moisture (%)**		**Total PAH (ng/g DWB)**	
		**Average**	**Range**	**Average**	**Range**
Hard pellet	2	3.9	3.5 – 4.4	210	193 – 227
Plug	1	19.3	Single sample	363	Single sample
Loose snus	10	56.5	54.1 – 57.7	399	283 – 737
Portion snus	22	48.0	9.6 – 55.9	434	269 – 898
Chewing tobacco	13	23.7	18.7 – 26.4	808	309 – 1251
Dry snuff	5	9.6	8.5 – 10.0	8651	573 – 11869
Moist snuff	16	54.2	50.0 – 56.2	10039	4151 – 19354
Soft pellet	1	17.3	Single sample	13972	Single sample

As expected, the different styles of STPs differed significantly in mean moisture contents. The highest moisture contents were found for the MS (54.2%) and snus (50.7%) styles. When the snus brands were separated into L and P styles the P snus had lower mean moisture (48%) than the loose snus (56.5%) mainly due to three of the P snus brands having particularly low moistures: Wise Citrus & Menthol Portion (9.6%), Catch Dry White Eucalyptus (25.9%) and Catch Dry White Licorice (27.5%). CT (23.7%), plug (19.3%) and SP (17.3%) had mean moistures that were much lower than MS or snus. DS (9.6%) and the HP product (3.9%) had the lowest moistures.

### Polycyclic aromatic hydrocarbons

The results for the PAH content of the STPs are shown in Additional file 2: Tables S3 and S4, on both a wet weight basis (WWB) and dry weight basis (DWB). The STPs are ordered by country of origin and product style.

#### Variation of PAHs with product style

All 38 US brands of STPs and most of the 32 Swedish snus brands contained the 21 PAHs measured, except for non-quantifiable levels of PER for 16 of the Swedish snus brands and of DB[*ah*]A for 30 of the Swedish snus brands.

##### Total PAHs

**Figure 3:**
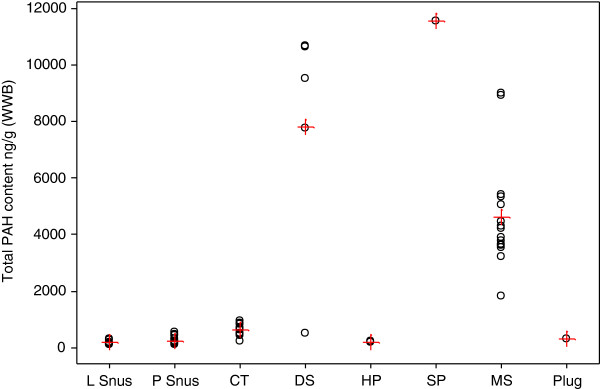
**Total PAH (means and individual concentrations, ng/g WWB) by product style.** Individual values are represented by open black circles, means by red crosses.

The mean values and ranges of total PAH concentrations (WWB) for the different styles of STPs are given in Additional file 3: Table S5 and also shown in Figure 3.

There were large variations in total PAH concentrations both between and within the product styles. Overall there was an almost 60 fold difference in mean concentrations between styles with the lowest (L snus, 173 ng/g WWB) and highest (SP, 11,555 ng/g WWB) mean concentrations of PAHs. Three product styles were associated with the highest levels of PAHs - SP, DS (7831 ng/g WWB) and MS (4621 ng/g WWB). These categories had levels of PAHs that were at least an order of magnitude greater than the other categories (CT, plug, L snus, P snus and HP).

Within the snuff and SP categories, analysis of variance (ANOVA) showed that the mean PAH concentrations of the SP and DS products were not significantly different from each other but were significantly higher than the PAH concentration in the MS product.

The differences in mean total PAH concentrations between the other categories of STP (CT, 615 ng/g WWB, L snus 173 ng/g WWB, P snus, 231 ng/g WWB, plug 293 ng/g WWB and HP 202 ng/g WWB) were not significant.

**Figure 4:**
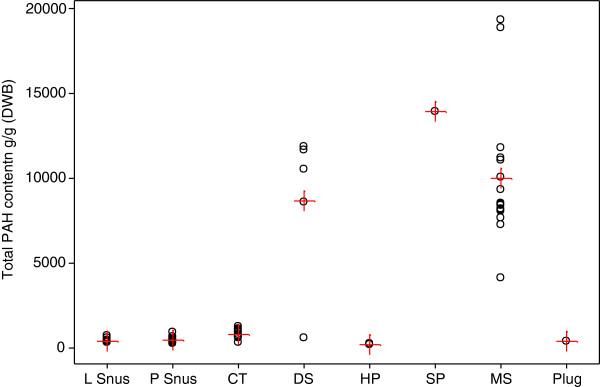
**Total PAH (means and individual concentrations, ng/g DWB) by product style.** Individual values are represented by open black circles, means by red crosses.

After correction for moisture, total PAH concentrations (Table 2 and Figure 4) were lowest for the HP products (210 ng/g DWB), and highest for the SP product (13,972 ng/g DWB). On a dry weight basis the MS category had a higher mean total PAHs (10,039 ng/g DWB) than the dry snuff category (8651 ng/g DWB). However the differences in total PAHs between the MS, DS and SP products were not significant.

##### Contributions of individual PAHs to total PAH concentrations

The mean absolute WWB concentration, DWB concentration and percentage contributions of the individual PAHs to total PAHs by product style are shown in Additional file 4: Table S6, Tables 3 and 4 respectively. The percent contributions of individual PAHs to the total are unchanged by moisture correction, as the same conversion factor is used to change WWB to DWB for each PAH within an STP sample.

**Table 3:** Contributions of the individual PAH to the totals for each product type (ng/g DWB)

	**Average PAH concentration for each product type (ng/g DWB)**							
	**Loose snus**	**Portion snus**	**Chewing tobacco**	**Dry snuff**	**Hard pellet**	**Soft pellet**	**Moist snuff**	**Plug**
**2-Ring**								
Naphthalene	96.6	112	54.0	84.9	69.7	76.5	110	53.4
1-methylnaphthalene	32.2	43.0	20.1	75.2	19.2	74.2	62.0	18.3
2-methylnaphthalene	17.6	27.0	10.4	60.0	10.5	65.6	46.9	9.51
**3-Ring**								
Acenaphthylene	4.40	6.68	5.3	62.6	4.79	81.6	52.9	2.68
Acenaphthene	4.86	8.65	3.4	49.5	5.31	79.6	48.3	4.39
Fluorene	21.8	29.3	15.6	429	21.3	695	437	8.89
Phenanthrene	73.0	76.3	207	2925	40.0	4541	3339	71.1
Anthracene	10.1	11.6	38.6	625	7.58	1210	731	13.3
**4-Ring**								
Fluoranthene	57.7	47.7	183	1512	9.84	2550	1854	67.9
Pyrene	42.8	36.6	163	1520	10.6	2519	1856	63.9
Benzo[*a*]anthracene	7.57	6.85	32.9	456	2.60	832	545	14.8
Chrysene	12.6	11.8	37.1	468	3.57	789	543	17
**5-Ring**								
Benzo[*b*]fluoranthene	3.36	3.03	7.3	74.2	0.938	97.6	80.0	3.35
Benzo[*k*]fluoranthene	2.06	1.95	3.7	32.3	0.644	38.4	34.0	1.87
Benzo[*j*]fluoranthene	2.48	2.39	5.9	55.9	0.688	65	57.9	2.89
Benzo[*e*]pyrene	2.50	2.26	6.0	65.8	0.821	77.4	67.7	2.8
Benzo[*a*]pyrene	2.93	2.53	6.0	80.4	0.971	117	87.4	3.25
Perylene	0.715	0.70	1.1	11.2	NQ	13.7	11.1	0.307
Dibenz[*a*,*h*]anthracene	NQ	0.55	0.5	5.7	BDL	5.67	6.67	NQ
**6-Ring**								
Indeno[*1*,*2*,*3*-*cd*]pyrene	2.00	1.47	3.6	31.0	0.452	26.1	35.8	1.59
Benzo[*ghi*]perylene	2.03	1.47	3.4	27.9	0.479	17.4	33.7	1.47
**TOTAL**	**399**	**434**	**808**	**8651**	**210**	**13972**	**10039**	**363**

NQ = Not quantified.

BDL = Below detection limit.

Based on average concentrations within each product type.

**Table 4:** Percentage contribution of individual PAH to the totals for each product type

	**Contribution of individual PAH to total (%)**							
	**Loose snus**	**Portion snus**	**Chewing tobacco**	**Dry snuff**	**Hard pellet**	**Soft pellet**	**Moist snuff**	**Plug**
**2-Ring**								
Naphthalene	24.2	25.8	6.7	1.0	33.2	0.5	1.1	14.7
1-methylnaphthalene	8.1	9.9	2.5	0.9	9.1	0.5	0.6	5.0
2-methylnaphthalene	4.4	6.2	1.3	0.7	5.0	0.5	0.5	2.6
**3-Ring**								
Acenaphthylene	1.1	1.5	0.7	0.7	2.3	0.6	0.5	0.7
Acenaphthene	1.2	2.0	0.4	0.6	2.5	0.6	0.5	1.2
Fluorene	5.5	6.8	1.9	5.0	10.2	5.0	4.3	2.5
Phenanthrene	18.3	17.6	25.6	33.8	19.0	32.5	33.3	19.6
Anthracene	2.5	2.7	4.8	7.2	3.6	8.7	7.3	3.7
**4-Ring**								
Fluoranthene	14.5	11.0	22.7	17.5	4.7	18.3	18.5	18.7
Pyrene	10.7	8.4	20.1	17.6	5.0	18.0	18.5	17.6
Benzo[*a*]anthracene	1.9	1.6	4.1	5.3	1.2	6.0	5.4	4.1
Chrysene	3.2	2.7	4.6	5.4	1.7	5.6	5.4	4.7
**5-Ring**								
Benzo[*b*]fluoranthene	0.8	0.7	0.9	0.9	0.4	0.7	0.8	0.9
Benzo[*k*]fluoranthene	0.5	0.4	0.5	0.4	0.3	0.3	0.3	0.5
Benzo[*j*]fluoranthene	0.6	0.6	0.7	0.6	0.3	0.5	0.6	0.8
Benzo[*e*]pyrene	0.6	0.5	0.7	0.8	0.4	0.6	0.7	0.8
Benzo[*a*]pyrene	0.7	0.6	0.7	0.9	0.5	0.8	0.9	0.9
Perylene	0.2	0.2	0.1	0.1	NQ	0.1	0.1	0.1
Dibenz[*a*,*h*]anthracene	NQ	0.1	0.1	0.1	BDL	0.0	0.1	NQ
**6-Ring**								
Indeno[*1*,*2*,*3*-*cd*]pyrene	0.5	0.3	0.4	0.4	0.2	0.2	0.4	0.4
Benzo[*ghi*]perylene	0.5	0.3	0.4	0.3	0.2	0.1	0.3	0.4
**TOTAL**	**100**	**100**	**100**	**100**	**100**	**100**	**100**	**100**

NQ = Not quantified.

BDL = Below detection limit.

Based on average concentrations within each product type.

#### MS, DS and SP

MS, DS and SP had, on average, the highest concentrations (WWB) of all individual PAHs except NAP, for which comparable concentrations were found for all the styles. Concentrations of the 3-6 ring PAHs were 5-15 fold higher in MS, DS and SP than in any of the other styles. Among the three styles, DS had greater WWB concentrations of all individual PAHs than MS. The SP product had greater concentrations of the 3-5 ring PAHs than MS or DS except for DB[*ah*]A. For all three styles PHEN was the greatest single contributor to total PAHs, accounting for approximately one third of the total.

After correction to a dry weight basis, MS, DS and SP still had the highest concentrations of all individual PAHs except NAP. However, the higher moisture of MS (mean 54.2%) vs DS (mean 9.6%) increased the calculated relative concentrations of PAHs in MS vs DS after moisture correction. This resulted in the DS having lower mean DWB concentrations than MS for all 3-6 ring PAH except ANY and ANE. These differences, however, were not significant.

#### CT and Plug

CT and plug had lower WWB concentrations of all the PAHs compared to those in the snuffs and SP. Compared with the snus and HP products, CT and plug had higher concentrations of PHEN, ANTH and the 4-6 ring PAH. The plug product had lower WWB concentrations of PHEN and the 4-6 ring PAHs than the CTs. PHEN was the single largest contributor (25.6%) to total PAHs in CT but there were also large contributions from FLNT (22.7%) and PYR (20.1%). For the plug product the largest contributions to total PAHs were also from PHEN (19.6%), FLNT (18.7%) and PYR (17.6%) but NAP (14.7%) also made a significant contribution.

Due to the relatively low moisture contents of the CTs (23.7%) and plug (19.3%) compared with the loose snus (56.5%) and P snus (48.0%), correction of the PAH concentrations to a DWB resulted in the concentrations of the 4-6 ring PAHs in the plug product being not significantly different to those in the snus and HP products. However, compared with either type of snus and HP, CT still had significantly higher DWB concentrations of all 4-6 ring PAHs as well as PHEN and ANTH.

#### Snus and HP

Loose snus had lower WWB concentrations of NAP, 1-MN and 2-MN than P snus, and the differences were significant for NAP and 2-MN. These differences were not changed after expressing the results on a DWB. For both types of snus, NAP (25%) was the greatest contributor to total PAHs, and contributions from PHEN (18%) and FLNT (12.8%) were much lower than for the snuffs and CT. For the HP category, NAP was also the greatest contributor and accounted for 33.2% of total PAHs.

Of all the categories of STPs the HP products had the lowest WWB and DWB concentrations of the 4-6 ring PAHs. For the 2- and 3-ring PAH the HP products were only lowest for PHEN and ANTH. WWB concentrations of the 2-ring PAHs and ANY, ANE and FLN were slightly higher (but not significantly) than those in snus, CT and plug. The relative concentrations of the PAH were not greatly changed by conversion to DWB.

### Naphthalene (NAP)

**Figure 5:**
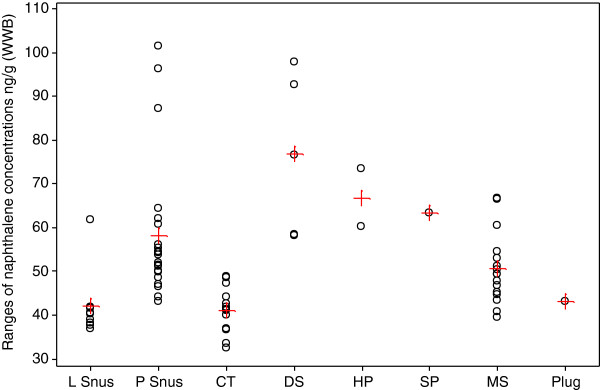
**Ranges of naphthalene concentrations (ng/g WWB) for the individual brands by product style.** Individual values are represented by open black circles, means by red crosses.

**Figure 6:**
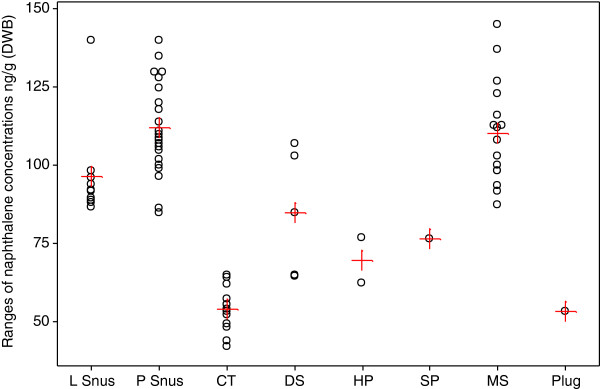
**Ranges of naphthalene concentrations (ng/g DWB) for the individual brands by product style.** Individual values are represented by open black circles, means by red crosses.

Concentrations of NAP and to a lesser extent 1-MN and 2-MN had quite different patterns across the different product styles, compared with the other PAHs. Figures 5 and 6 illustrate the ranges and mean NAP concentrations of the individual brands by product style, on a WWB and DWB respectively.

Compared with the total PAH concentrations (Figure 3), NAP concentrations had a more limited range of values. The highest concentrations (WWB) were found in DS (average 76.8 ng/g), but these concentrations were not significantly different to those in HP (67 ng/g), SP (63.3 ng/g) and plug (43.1 ng/g). Significantly lower concentrations were found in P snus (58.2 ng/g), MS (50.6 ng/g), L snus (42 ng/g) and CT (41.2 ng/g) products. After correction to DWB, P snus (112 ng/g) and MS (110 ng/g) had the highest mean concentrations of NAP and these were significantly higher than those in any other product categories. Accounting for possible dilution effects of sugar, glycerol and propylene glycol amongst the different products further reduced, but did not eliminate, differences between the product categories (e.g. loose and portion snus products still had different PAH contents).

The low total PAH concentrations for the snus and HP products (Table 2) resulted in NAP being the most abundant PAHs for these styles with contributions of 25% and 33% respectively (Table 4). The reasons for the anomalous results for NAP, with relatively higher concentrations in those products with low total PAH concentrations, are unknown. Selective loss of the more volatile NAP compared with the other PAHs during processing is not consistent with, for example, the high temperatures used to pasteurise snus, although losses of NAP during product storage cannot be ruled out. Other possible explanations include lower levels of NAP in the fire-cured tobaccos that contribute to the higher molecular weight PAHs, or other, as yet unidentified, sources of NAP, to which the products may have been exposed.

The highest mean concentrations of 1- and 2-MP were found in the DS and SP product(s). The lowest concentrations of the methyl naphthalenes were found in the L snus, CT, HP and plug products.

### Correlations between PAHs in STPs

The correlations between the concentrations of the different PAHs on a DWB basis were calculated using Minitab Version 16. The matrix of the Pearson correlations and P-values are given in Additional file 5: Table S7.

Naphthalene correlated poorly with all the other PAHs measured, the highest correlation (r = 0.403) being with 1-MP. 1- and 2-MP correlated highly with each other (r = 0.992), but the correlations with the other PAHs decreased as the PAH size increased, down to r = 0.62, for example, for 1-MP with B[*g,h,i*]P. The larger PAHs correlated well with each other, with PAHs larger than PHEN having correlations of greater than 0.93. In particular, B[*a*]P had correlation coefficients greater than 0.9 for all the PAHs measured except for NAP and 1- and 2- MP. Hence its use as a marker for levels of PAHs in STPs appears to be justified.

## Discussion

This study is the most extensive survey of PAHs in STPs published to date. The concentrations of 21 PAHs in 70 brands of STPs, covering the major STP categories sold in the US and Sweden were examined. A wide variation in PAH contents was observed across different STP categories. High PAH concentrations in the snuffs and SP product are consistent with the reported relatively large proportions of fire-cured tobaccos used in these product styles. Other styles of smokeless tobacco are reported to use little or no fire-cured tobacco [19], and were shown in this work to contain much lower levels of PAHs. Use of fire-cured tobacco was phased out in snus production during the 1990′s, and B[*a*]P (analysed as a proxy for PAHs) dropped from 20-25 ng/g DWB to less than 2-3 ng/g DWB in the period 1998-2004 [13].

### Comparison of results with earlier studies

The concentrations of very few PAHs other than B[*a*]P have been reported in the literature. For B[*a*]P there have been several published reports covering B[*a*]P concentrations in MS and DS, HP, snus, spit-free and Asian products. These [8,10-13,20-22] are shown in Table 5 together with a summary of the results from this study.

**Table 5:** Literature values for B[a]P in smokeless products

**Product category**	**Country**	**B[a]P this study (ng/g DWB)**	**B[a]P literature values (ng/g DWB)**	
CT	US	2.72 – 10.6	BDL–NQ	[11]
			1.2 – 8	[21]
DS	US	6.1 – 122	0.1 – 90	[8]
			0.7–118	[21]
HP	US	0.66 – 1.28	0.4	[10]
			0.3–0.4	[21]
MS	US	35.8 – 167	0.1 – 63	[8]
			19.3	[10]
			30.1–57.3	[22]
			21.1–83.2	[11]
			13–102	[12]
			0.6–193	[21]
Plug	US	3.3	5.4	[21]
Snus	Swedish	1.58 – 5.23	1.99	[10]
			1.59–2.08	[11]
			1.1 average	[13]
			0.3–4.1	[21]
SP	US	117	1.8–88.5	[21]
Miscellaneous	India	N/A	0.1–940	[23]
**Other styles of STP**				
Masheri^1^	India	N/A	27.0 – 119	[9]
Spit Free^2^	US	N/A	<1.02–10.5	[22]
			<1.6–15.6	[12]
Dry Snuff	UK	N/A	11.8 – 18.6	[11]
Gutkha^3^	UK & Asia	N/A	0.4–1.28	[10]
			18.3	[11]
Zarda^4^	Asia	N/A	0.32–8.89	[10]

^1^Masheri or mishri is a pyrolysed and powdered tobacco used in India.

^2^Spit-free is a US version of portion snus.

^3^Gutkha is a sweet chewing tobacco containing betel leaf, catechu & saffron.

^4^Zarda is a moist or dry chewing tobacco mixed with colourings, spice essences, & perfumes.

*N/A* Not available, *NQ* Not quantified, *BDL* Below detection limit.

Results from the present study ranged from 0.7 to 167 ng/g (DWB) compared with ranges in the literature from 0.1 - 193 ng/g (DWB). Of all the studies, the most recent [20] reported B[*a*]P concentrations in the widest range of product styles and brands, including different styles of US STPs and Swedish snus. Their results bracket the results found in the earlier studies and are in good agreement with the present study.

There have only been two studies, to date, that have reported levels of PAHs other than B[*a*]P in smokeless tobacco products on the US market [12,21], and the only published data available for Swedish products reports the content of one product [21].

The list of PAHs determined in the current and the two earlier studies is shown in Table 6. The table also includes those PAHs on the FDA Established List of Hazardous and Potentially Hazardous Chemicals (HPHC), as well as the IARC classification of carcinogenicity for each of the PAHs.

**Table 6:** Polycyclic aromatic hydrocarbons reported in STPs and those included in the FDA HPHC established list

	**IARC classification**^**1**^	**FDA HPHC established List **[3]	**Current study**	**Stepanov et al. **[21]	**Stepanov et al. **[12]
Acenaphthene	3		yes		yes
Acenaphthylene	Not listed		yes	yes	yes
Anthracene	3		yes	yes	yes
Benz[*a*]anthracene	2B	yes	yes		yes
Benz[*j*]aceanthrylene	2B	yes			
Benzo[*a*]pyrene	1	yes	yes	yes	yes
Benzo[e]pyrene	3		yes		yes
Benzo[*b*]fluoranthene	2B	yes	yes	yes^5^	yes^2^
Benzo[*c*]phenanthrene	2B	yes			
Benzo[*j*]fluoranthene	2B		yes		yes^2^
Benzo[*k*]fluoranthene	2B	yes	yes	yes^5^	yes
Benzo[*ghi*]perylene	3		yes		yes
Chrysene	2B		yes		yes
1-methylchrysene	3				yes^4^
3-methylchrysene	3				yes^4^
4- & 6-methylchrysene	3				yes^4^
5-methylchrysene	2B	yes			yes^3,4^
Cyclopenta[*c*,*d*]pyrene	2A	yes			
Dibenz[*a*,*h*]anthracene	2A	yes	yes		yes
Dibenzo[*a*,*e*]pyrene	2B	yes			
Dibenzo[*a*,*h*]pyrene	2B	yes			
Dibenzo[*a*,*i*]pyrene	2B	yes			
Dibenzo[*a*,*l*]pyrene	2A	yes			
Fluoranthene	3		yes	yes	yes
Fluorene	3		yes		yes
Indeno[*1*,*2*,*3*-*cd*]pyrene	2B	yes	yes		yes
Naphthalene	2B	yes	yes		yes
1-methylnaphthalene	Not listed		yes		
2-methylnaphthalene	Not listed		yes		
Perylene	3		yes		
Phenanthrene	3		yes	yes	yes
Pyrene	3		yes	yes	yes

^1^IARC class key: Group 1: Carcinogenic to humans, Group 2A: Probably carcinogenic to humans, Group 2B: Possibly carcinogenic to humans, Group 3: Not classifiable as to carcinogenicity to humans.

^2^Benzo[*b*]fluoranthene and benzo[*j*]fluoranthene were not separated.

^3^5-methyl chrysene was not detected in any of the samples.

^4^methylchrysenes reported as “total methylchrysenes”.

^5^Benzo[*b*]fluoranthene and benzo[*k*]fluoranthene were not separated.

The earlier (2008) study by Stepanov et al. [21] reported the concentrations of 8 PAHs in 16 US smokeless tobaccos - 12 US portion snus products, 4 MSs and one Swedish snus. The more recent (2010) study [12] examined 23 MS brands and 17 US portion snus products for the presence of 23 PAHs, and identified the presence of 22 PAHs in their survey samples. There was some overlap in products and PAHs between the two Stepanov et al. studies, and there were substantial differences in reported levels for many of the brands and PAHs common to both studies. For example levels of individual PAHs in the MS samples ranged from 2- to 10-fold lower for the same brand in the 2008 study compared to the results reported in 2010.

The more recent study by Stepanov et al. [12] also included PAHs and MS brands that were common to the present study, so we had the opportunity to compare our results with those of Stepanov et al. This will be shown in the next section.

### Comparison of PAH concentrations in current and historic samples

**Figure 7:**
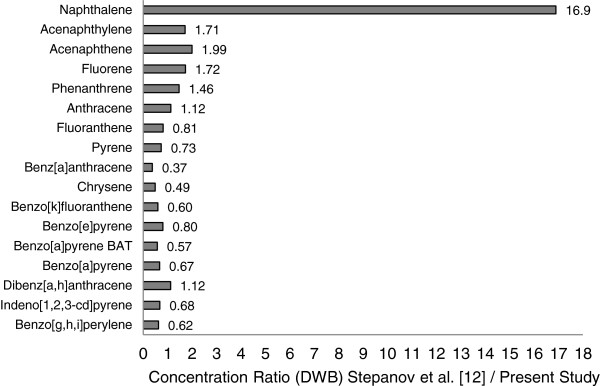
**Ratio of PAH levels reported by Stepanov et al.**[12]**to those obtained in the present study.**

To compare the consistency in STP PAH concentrations between the study of Stepanov et al. [12] and the present study, the mean concentrations of the PAHs and products common to both studies were calculated on a DWB. The nine brands of MS common to both studies were: Copenhagen LC, Grizzly Natural LC, Kayak Straight LC, Kodiak Straight LC, Kodiak Wintergreen, Skoal Straight, Timberwolf Natural FC, Timberwolf Straight LC and Red Seal Natural FC. The ratios of these results were calculated for each of the PAHs that were measured in both studies. The ratios are plotted in Figure 7. Values close to 1 indicate good agreement, with values below 1 indicating that the results from the present study were higher than those reported by Stepanov et al. and values greater than 1 indicating that the Stepanov et al. results were higher.

For the majority of the PAHs the ratios of the results reported by Stepanov et al. [12] and the values obtained for the same products within the present study, were between 0.5 and 2.0. Given the likelihood of batch to batch variability in the products and differences in methodology between the two laboratories these ratios can be considered as indicating good agreement between the studies. However, the results for NAP were markedly different. Stepanov et al. found that NAP was the greatest contributor to total PAH levels in all of the samples, and their reported levels were almost 16 times higher than those found in the present study.

There is no clear explanation for these differences, but given that the differences in NAP concentrations are so substantial, and that NAP is an established FDA HPHC, further investigation should be a priority for future research into STP toxicant chemistry.

### Sources of PAH in STPs

As noted earlier, MS and DS contain significant levels of fire-cured tobaccos which have been identified as major sources of PAHs [12] and our findings of high levels of PAHs in these STP categories are consistent with this. The relatively high levels of PAHs that we found in the SP product also suggest the incorporation of substantial amounts of fire-cured tobacco in this STP.

The use of fire-cured tobaccos in snus was abandoned by Swedish Match in the 1990s [13], and this is reflected in the low levels of PAHs in snus products from Swedish Match (and from the other snus manufacturers). The other STPs (HP, Plug, and CT) also have low PAHs relative to DS, MS and SP. Therefore the reasons for the presence, albeit at low levels, of PAHs in snus and other non-fire-cured tobacco containing STPs is unclear. Rickert et al. [11] postulated that the presence of B[*a*]P in non-fire-cured STPs may arise from sources such as environmental contamination of the leaf surfaces or inadvertent exposure to combustion fumes during processing. In an attempt to pinpoint the possible sources of PAHs more clearly we have examined the ratios of PAHs within the different classes of STPs. A number of researchers have noted that different combustion sources, including domestic and industrial wood or coal combustion, natural or agricultural fires, anode baking in the aluminium industry, and gasoline and diesel powered vehicles, produce PAHs with different relative abundances. The ratios of individual PAHs have been used to identify their sources in a range of products such as vegetables, fish and coffee [6]. This approach was used in the current study to understand possible sources of PAHs in the different STP categories.

The ratio ANTH/(ANTH + PHEN) has been proposed as a means of distinguishing between low temperature (petroleum combustion) sources or higher temperature (wood combustion) sources, with a ratio <0.1 indicating petroleum sources and a ratio >0.1 indicating mainly higher temperature combustion sources of PAHs [23-27]. In the present study the ANTH/(ANTH + PHEN) ratio for both snus categories covered a range from below 0.1 to around 0.15, with a mean value of approximately 0.13. In contrast the ratios for DS, MS and SP were distinctly higher, ranging from 0.16 to 0.22. Ratio values for HP, Plug and CT were intermediate between these two groups. These observations suggest differences in the sources of PAHs between these category groups, with higher temperature combustion sources dominating with DS, MS and SP, and mixed sources, including lower temperature petrogenic sources, generating the PAH content of snus products.

Similar conclusions were reached with the ratio B[*a*]A/(B[*a*]A + CHR). Hischenhuber and Strijve [28] suggested that B[*a*]A/(B[*a*]A + CHR) values <0.2 involve petroleum combustion, ratios from 0.2-0.35 indicate either petroleum or higher temperature (wood or coal) combustion, and ratios >0.35 result from higher temperature combustion processes. In this study values for both snus categories and HP products ranged from <0.35 to 0.45, whereas plug, CT, DS, MS and SP had ratios from 0.42-0.52, implying mixed but predominately higher temperature combustion sources for snus and HP, and high temperature combustion as the exclusive source of PAH for the other STP categories.

In environmental matrices (e.g. sediments, organisms or air) FLNT/(FLNT + PYR) values >0.5, indicate sources including grass, wood or coal combustion, whereas FLNT/(FLNT + PYR) <0.4 indicate gasoline, diesel and fuel oil combustion [23-25,29]. Substantial differences were found in the FLNT/(FLNT + PYR) ratios between PS, LS and HP products (mean within category ratios of 0.55, 0.55 and 0.65 respectively), and the group of STP categories comprising CT (mean within category FLNT/(FLNT + PYR) = 0.1), plug (0.12), DS (0.2), MS (0.18) and SP (0.22). While the differences between categories in this work are clear and distinct, it is difficult to reconcile the known presence of wood combustion products in DS, MS and SP with the sources indicated by the FLNT/(FLNT + PYR) ratio. The indication of grass, wood or coal combustion as the sources of PAHs with snus and HP products may indicate environmental contamination from agricultural fires or from domestic and industrial heating sources.

**Figure 8:**
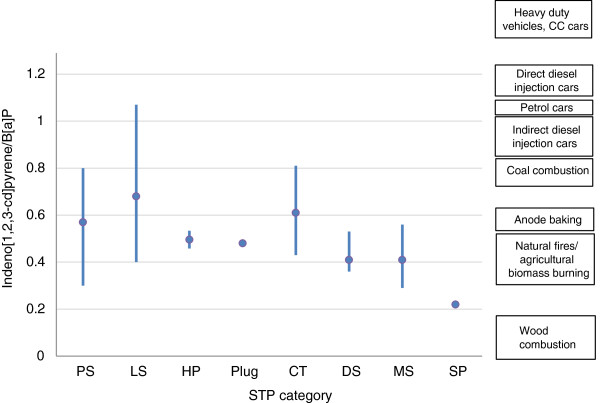
**Comparison of STP Indeno[*1,2,3-cd*]pyrene/B[*a*]P ratios for different STP categories with literature values for potential sources.**

The measured ratios of I[*cd*]P/B[*a*]P amongst STP categories, together with indications of origin [30], are compared in Figure 8. The ratios for DS, MS and SP indicate sources such as natural fires/agricultural biomass and wood combustion, consistent with the use of fire-cured tobaccos in these STPs. In contrast, mean values for PS, LS, CT and Plug are higher, and consistent with mixed sources but with strong contributions from petrogenic, coal combustion and/or anode baking sources. The range of values for both snus categories is wider than with the other categories, and analysis by manufacturer showed systematic differences suggesting different sources of tobacco (leading to differences in PAH profiles) amongst the snus manufacturers.

**Figure 9:**
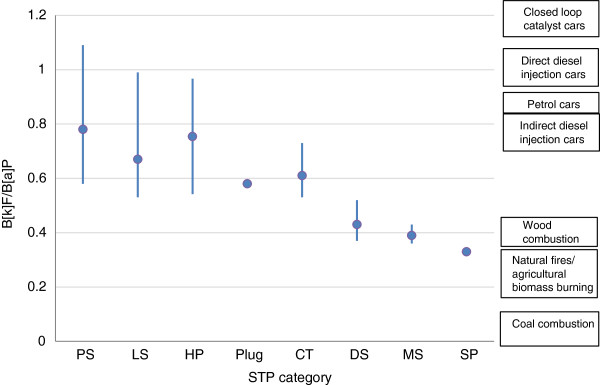
**Comparison of STP B[*k*]F/B[*a*]P ratios with literature values for potential sources.**

The ratio B[*k*]F/B[*a*]P (Figure 9) also showed significant differences between the group comprising PS, LS, HP, Plug and CT, and the group comprising DS, MS and SP. Once again, the latter group showed B[*k*]F/B[*a*]P values indicating [30] wood combustion and natural fires/agricultural biomass burning (consistent with fire-cured tobacco use in this group). The STP group consisting of snus, CT, Plug and HP showed B[*k*]F/B[*a*]P ratios consistent with mixed sources of PAHs, but with strong contributions from PAHs from petrogenic sources. The wide range of values for both snus products showed differences between manufacturers with some (Skruff, Habaneros, Northerner) using tobaccos with dominant contribution from petrogenic PAHs, and others (Swedish Match, Fielder & Lundgren, and Japan Tobacco International) using tobaccos with stronger contributions from wood combustion sources.

Finally, the B[*b*]F/B[*a*]P ratio was also compared amongst STP categories. This ratio was found to be less informative due to overlap and similarity in ratios between petrogenic, wood and agricultural biomass combustion sources [30]. However, the B[*b*]F/B[*a*]P ratio for coal combustion is substantially lower than those found for petrogenic and wood/biomass combustion sources, and inspection of the B[*b*]F/B[*a*]P ratios showed that none of the STP categories had evidence of any significant contribution to their PAH loadings from coal combustion.

**Figure 10:**
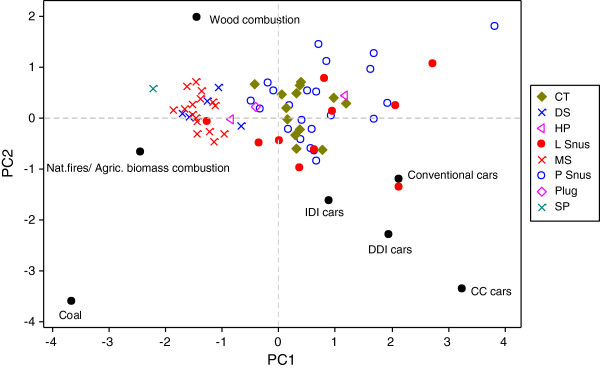
**PCA analysis of STP PAH ratios in comparison to literature values for likely sources with potential sources.** Abbreviations in the figure: IDI = indirect diesel injection car engines, DDI = direct diesel injection car engines, CC = closed loop catalytic car engines.

A principal component analysis (PCA) of the I[*cd*]P/B[*a*]P, B[*k*]F/B[*a*]P and B[*b*]F/B[*a*]P ratios for all of the STPs and the likely PAH sources [30] is shown in Figure 10. Figure 10 clearly shows that the main contributors of PAH levels in DS, MS and SP are wood combustion together with natural fires/agricultural biomass, i.e. fire-curing. The data for these three STP categories are tightly gathered on the PCA plot. In contrast the snus products cover a very wide area of the PCA space, and show a clear influence from petrogenic sources such as diesel and petrol cars, in addition to wood/biomass combustion. CT and Plug cover a similar space to the snus products, whereas the two HP products show diverse profiles. It is notable that coal combustion shows little contribution to the PAH profiles of the studied STPs, and neither do closed loop catalyst cars.

In conclusion, examination of a number of diagnostic PAH ratios for the STPs measured in this work showed that the relatively high levels found with SP, DS and MS clearly arise from relatively high temperature processes involving wood and agricultural biomass combustion sources. This is consistent with the known use of fire-cured tobaccos in US snuff products.

Much lower levels of PAHs were found in snus, but their source was both more diverse and highly dependent upon manufacturer, implying differences in geographical sourcing of tobaccos. Lower temperature petrogenic sources were found to be important contributors to PAHs in snus, along with contributions from higher temperature combustion sources such as wood, and agricultural biomass combustion as well as natural fires. The relative contribution of these sources varies among snus products, resulting from exposure to varying environmental pollution sources generating the PAHs. Whereas reduction in PAH levels in STPs containing fire-cured tobaccos could be achieved by tobacco blending choices, the plurality of low level environmental PAH sources with snus suggests that control and reduction in PAH levels beyond their current relatively low levels may be a challenging exercise dependent upon successfully minimising the impact of multiple general societal factors.

## Conclusions

In this study we have quantified the levels of 21 PAHs in a wide range of both US and Swedish smokeless tobaccos. We report for the first time the levels of 1-MN, 2-MN and PER in smokeless tobacco. Together with the 22 PAHs previously quantified in smokeless tobacco by Stepanov et al. [12] our study brings the total number of quantified PAHs in STPs to 25. These are classified (by IARC carcinogenicity) as one Group 1, one Group 2A, eight Group 2B, twelve Group 3 and three unclassified. Several of the FDA HPHC PAHs - benz[*j*]aceathrylene, benzo[*c*]phenanthrene, cyclopenta[*c*,*d*]pyrene and four dibenzopyrenes - have yet to be quantified in STPs, and this represents a further research need for fuller characterisation of toxicants in STPs.

This study is also the first in which PAHs (other than B[*a*]P) have been determined in an extensive range of Swedish snus products. It was found that total concentrations of PAHs in US SP, MS and DS were, on average, 10-60 fold greater than those in Swedish snus and in US HP, CT, and plug. The HP products had the lowest total concentrations of PAHs. Of the individual PAHs, those with higher molecular weights (3-6 ring) had similar concentration patterns to total PAHs across the different STPs, with PHEN, FLNT and PYR having the highest concentrations. For NAP, however, the range of concentrations was much lower than for the other PAHs, and Swedish snus products, on average, had comparable concentrations to those of MS and DS, and slightly higher than those in CT. The HP products had the lowest concentrations of the majority of individual PAHs.

The excellent correlation between B[*a*]P and the 3-6 ring PAHs means that B[*a*]P can be used reliably as a marker for these PAHs in STPs. Naphthalene correlated poorly with B[*a*]P and would have to be measured separately in a general assessment of PAH concentrations.

Generally good agreement was found between our results and those of a previous study of PAH content of STPs except for gross differences in reported NAP concentrations. Given the presence of NAP amongst 15 PAHs on the FDA HPHC list, there is an urgent need to develop analytical methods that will provide more robust and consistent data across different laboratories and studies.

The high concentrations of PAHs in MS, DS and SP are consistent with their blends containing large proportions of fire-cured tobaccos, as has been described previously. The sources of the much lower levels of PAHs in the other styles of STPs have not been identified historically, but analysis in this study of the ratios of different individual PAHs including principal component analysis suggests that there are a variety of contributors to the trace levels of PAHs in these STP categories, with predominant contributions from petrogenic and combustion sources.

## Abbreviations

ANE: Acenaphthene; ANTH: Anthracene; ANY: Acenaphthylene; B[a]A: Benzo[*a*]anthracene; B[a]P: Benzo[*a*]pyrene; B[b]F: Benzo[*b*]fluoranthene; B[e]P: Benzo[*e*]pyrene; B[ghi]P: Benzo[*ghi*]perylene; B[j]F: Benzo[*j*]fluoranthene; B[k]F: Benzo[*k*]fluoranthene; BLD: Below the limit of detection; CHR: Chrysene; CORESTA: Cooperation centre for scientific research relative to tobacco; CT: Chewing tobacco; DB[ah]A: Dibenz[*a*,*h*]anthracene; DS: Dry snuff; DWB: Dry weight basis values; the value once corrected for moisture content; FDA: US Food and drug administration; FLN: Fluorene; FLNT: Fluoranthene; HP: Hard pellet; HPHC: Harmful and potentially harmful constituents; IARC: International agency for research in cancer; I[cd]P: Indeno[1,2,3-cd]pyrene; L snus: Loose snus; 1-MN: 1-methylnaphthalene; 2-MN: 2-methylnaphthalene; MS: Moist snuff; NAP: Naphthalene; NQ: Not quantified; PAH: Polycyclic aromatic hydrocarbon; PER: Perylene; PHEN: Phenanthrene; P snus: Portion snus; PYR: Pyrene; SP: Soft pellet; STP: Smokeless tobacco product; WWB: Wet weight basis values; the value as measured on the STP in its natural form.

## Competing interests

KM and HK work for a tobacco company, as did AF at the time of the study. AP is a consultant to British American Tobacco. BR’s research is supported by unrestricted grants from tobacco manufacturers (including BAT) to the University of Louisville, and by the Kentucky Research Challenge Trust Fund.

## Authors’ contributions

KM directed the research, jointly wrote the manuscript, and conducted the data analysis. AF and HK managed the measurement programme and assisted with data analysis, BR jointly directed the research and jointly wrote the manuscript, AP aided in the data analysis and preparation of the manuscript. All authors read and approved the final manuscript.

## Supplementary Material

Additional file 1: Table S1 Swedish smokeless products tested in the survey. **Table S2.** American smokeless products tested in the survey. Click here for file

Additional file 2: Tables S3 and S4 Contains the averages and standard deviations of the PAH concentrations (WWB and DWB respectively) and moisture contents for the STPs. Click here for file

Additional file 3: Table S5 Averages and ranges of total PAH concentrations (ng/g WWB) by product style. Click here for file

Additional file 4: Table S6 Contributions of the individual PAH to the totals for each product type (ng/g WWB). Based on average concentrations within each product type. Click here for file

Additional file 5: Table S7 Pearson coefficients and P-values for correlations between the PAH concentrations (DWB) in the STPs. Click here for file
