# Supplementary material for: Polycyclic aromatic hydrocarbons in US and Swedish smokeless tobacco products
Source: Chem Cent J. 2013 Sep 8;7:151. doi: 10.1186/1752-153X-7-151 (PMC3874832; doi:10.1186/1752-153X-7-151)
Supplement: Additional file 1: Table S1 — Swedish smokeless products tested in the survey. Table S2. American smokeless products tested in the survey. [file 1752-153X-7-151-S1.docx]

Table S1

Swedish smokeless products tested in the survey.

| Swedish Smokeless Products | Style | % Market Share (2008)^1^ | Manufacturer |
| --- | --- | --- | --- |
| General | L Snus | 8.27 | Swedish Match |
| Ettan |  | 7.84 |  |
| Grovsnus |  | 5.44 |  |
| Göteborgs Rapé |  | 2.30 |  |
| Kronan |  | 4.06 |  |
| Granit |  | 0.93 | Fiedler & Lundgren |
| LD |  | 0.14 | Japan Tobacco International |
| Skruf Strong |  | 0.20 | Skruf Snus AB |
| T. Montecristo |  | 0.02 | Habanos Nordic |
| Knox |  | 0.30 | Skruf Snus AB |
| General White | P Snus | 4.84 | Swedish Match |
| General, mini |  | 2.28 |  |
| General |  | 11.90 |  |
| Ettan |  | 4.29 |  |
| Grovsnus |  | 5.21 |  |
| Grovsnus White |  | 1.39 |  |
| Göteborgs Rapé White |  | 7.86 |  |
| Kronan |  | 5.44 |  |
| Catch Licorice, Mini |  | 2.16 |  |
| Catch White Licorice |  | 1.79 |  |
| Catch Dry White Eucalyptus, Mini |  | 1.46 |  |
| Catch Dry White Licorice, Mini |  | 1.22 |  |
| Granit White |  | 3.18 | Fiedler & Lundgren |
| Granit |  | 2.74 |  |
| Tre Ankare White |  | 1.64 | Swedish Match |
| LD Original |  | 0.63 | Japan Tobacco International |
| Level |  | 0.38 |  |
| Skruf Strong |  | 0.34 | Skruf Snus AB |
| Knox |  | 0.29 |  |
| Wise Citrus & Menthol (6 mg)^2^ |  | 0.02 | Njette |
| T. Romeo y Julieta |  | 0.02 | Habanos Nordic |
| 1847 Original |  | N/A^3^ | Philip Morris International |

^1^ Market share includes flavour variants of the same brand

^2^Subsequent to the purchase of this product ownership changed and the product was renamed Northerner Citrus & Menthol

^3^ N/A Not available

Table S2

American smokeless products tested in the survey

| American Smokeless Product | Category | % Market share of brand family within category (2008) | Manufacturer |
| --- | --- | --- | --- |
| Chattanooga | Chewing Tobacco | 2 | Swisher |
| Starr |  | 7 |  |
| Lancaster |  | 3 |  |
| Durango |  | 2 | National |
| BeechNut |  | 6 |  |
| Stoker 707 Wintergreen |  | 11 |  |
| Trophy |  | 3 |  |
| Levi Garrett |  | 18 | Conwood^1^ |
| Morgan’s |  | 3 |  |
| Taylors Pride |  | 4 |  |
| Red Man Gold |  | 13 | Swedish Match^2^ |
| Red Man Regular |  | 18 |  |
| Southern Pride |  | 4 |  |
| Bruton | Dry snuff | 10 | US Smokeless Tobacco |
| Dental Sweet |  | 7 | Conwood |
| Garrett |  | 21 |  |
| Honest |  | N/A^3^ (<9%) |  |
| Square |  | 4 | Swisher |
| Ariva Java | Hard Pellet | N/A | Star Scientific |
| Stonewall Wintergreen |  |  |  |
| Oliver Twist Original | Soft Pellet | N/A | House of Oliver Twist |
| Copenhagen LC | Moist Snuff | 24 | US Smokeless Tobacco |
| Copenhagen Straight LC |  |  |  |
| Husky Natural FC |  | <1% |  |
| Husky Straight LC |  |  |  |
| Husky Wintergreen |  |  |  |
| Skoal Straight |  | 25 |  |
| Red Seal Natural FC |  | 7 |  |
| Red Seal Natural LC |  |  |  |
| Kayak Straight LC |  | 2 | Swisher |
| Silver Creek |  | 1 |  |
| Grizzly Natural LC |  | 24 | Conwood |
| Kodiak Straight LC |  | 4 |  |
| Kodiak Wintergreen |  |  |  |
| Timberwolf Natural FC |  | 6 | Swedish Match |
| Timberwolf Straight LC |  |  |  |
| Marlboro Original LC |  | N/A | Phillip Morris |
| Cannonball | Plug | 33 | Conwood |

^1^ Conwood, a division of Reynolds American, was renamed American Snuff Company in 2010.

^2^ Swedish Match sells US products under the name Pinkerton Tobacco

^3^ N/A Not available
