# Supplementary material for: Polycyclic aromatic hydrocarbons in US and Swedish smokeless tobacco products
Source: Chem Cent J. 2013 Sep 8;7:151. doi: 10.1186/1752-153X-7-151 (PMC3874832; doi:10.1186/1752-153X-7-151)
Supplement: Additional file 3: Table S5 — Averages and ranges of total PAH concentrations (ng/g WWB) by product style. [file 1752-153X-7-151-S3.docx]

Additional Table S5

Averages and ranges of total PAH concentrations (ng/g WWB) by product style.

| Style | Country | # Brands | Total PAH (ng/g WWB) | |
| --- | --- | --- | --- | --- |
|  |  |  | Average | Range |
| Loose Snus | Sweden | 10 | 173 | 120 - 315 |
| Hard Pellet | US | 2 | 202 | 186 - 217 |
| Portion Snus | Sweden | 22 | 231 | 132 - 564 |
| Plug | US | 1 | 293 | Single sample |
| Chewing Tobacco | US | 13 | 615 | 236 - 921 |
| Moist Snuff | US | 16 | 4621 | 1818 - 9006 |
| Dry Snuff | US | 5 | 7831 | 516 - 10683 |
| Soft Pellet | US | 1 | 11555 | Single sample |
