# Supplementary material for: Polycyclic aromatic hydrocarbons in US and Swedish smokeless tobacco products
Source: Chem Cent J. 2013 Sep 8;7:151. doi: 10.1186/1752-153X-7-151 (PMC3874832; doi:10.1186/1752-153X-7-151)
Supplement: Additional file 4: Table S6 — Contributions of the individual PAH to the totals for each product type (ng/g WWB). Based on average concentrations within each product type. [file 1752-153X-7-151-S4.docx]

Additional Table S6

Contributions of the individual PAH to the totals for each product type (ng/g WWB). Based on average concentrations within each product type.

|  | PAH concentration for each product type (ng/g WWB) | | | | | | | |
| --- | --- | --- | --- | --- | --- | --- | --- | --- |
|  | Loose Snus | Portion Snus | Chewing  Tobacco | Dry  Snuff | Hard  Pellet | Soft Pellet | Moist  Snuff | Plug |
| **2-Ring** |  |  |  |  |  |  |  |  |
| Naphthalene | 42 | 58.2 | 41.2 | 76.8 | 67.0 | 63.3 | 50.6 | 43.1 |
| 1-methylnaphthalene | 14 | 24.4 | 15.3 | 68.1 | 18.4 | 61.4 | 28.5 | 14.8 |
| 2-methylnaphthalene | 7.6 | 16.2 | 7.9 | 54.3 | 10.1 | 54.3 | 21.6 | 7.7 |
| **3-Ring** |  |  |  |  |  |  |  |  |
| Acenaphthylene | 1.9 | 3.6 | 4.0 | 56.6 | 4.6 | 67.5 | 24.4 | 2.2 |
| Acenaphthene | 2.1 | 4.6 | 2.6 | 44.8 | 5.1 | 65.8 | 22.3 | 3.5 |
| Fluorene | 9.5 | 15.5 | 11.9 | 388 | 20.4 | 575 | 201 | 7.2 |
| Phenanthrene | 32 | 40.1 | 157 | 2648 | 38.3 | 3755 | 1538 | 57.4 |
| Anthracene | 4.4 | 6.1 | 29.4 | 565 | 7.3 | 1001 | 337 | 10.7 |
| **4-Ring** |  |  |  |  |  |  |  |  |
| Fluoranthene | 25 | 24.9 | 140 | 1369 | 9.5 | 2109 | 853 | 54.8 |
| Pyrene | 18.6 | 19.1 | 124 | 1376 | 10.2 | 2083 | 854 | 51.6 |
| Benzo[*a*]anthracene | 3.3 | 3.6 | 25 | 413 | 2.5 | 688 | 251 | 11.9 |
| Chrysene | 5.5 | 6.3 | 28.2 | 423 | 3.4 | 653 | 250 | 13.7 |
| **5-Ring** |  |  |  |  |  |  |  |  |
| Benzo[*b*]fluoranthene | 1.5 | 1.6 | 5.5 | 67.1 | 0.90 | 80.7 | 36.8 | 2.7 |
| Benzo[*k*]fluoranthene | 0.9 | 1.0 | 2.8 | 29.2 | 0.62 | 31.8 | 15.6 | 1.5 |
| Benzo[*j*]fluoranthene | 1.1 | 1.3 | 4.5 | 50.6 | 0.66 | 53.8 | 26.6 | 2.3 |
| Benzo[*e*]pyrene | 1.1 | 1.2 | 4.5 | 59.5 | 0.79 | 64.0 | 31.2 | 2.3 |
| Benzo[*a*]pyrene | 1.3 | 1.3 | 4.6 | 72.7 | 0.93 | 96.8 | 40.3 | 2.6 |
| Perylene | 0.3 | 0.37 | 0.80 | 10.1 | NQ | 11.3 | 5.1 | 0.26 |
| Dibenz[*a,h*]anthracene | NQ | 0.29 | 0.40 | 5.1 | BDL | 4.7 | 3.1 | NQ |
| **6-Ring** |  |  |  |  |  |  |  |  |
| Indeno[*1,2,3-cd*]pyrene | 0.9 | 0.8 | 2.8 | 28.0 | 0.43 | 21.6 | 16.5 | 1.3 |
| Benzo[*ghi*]perylene | 0.9 | 0.8 | 2.6 | 25.3 | 0.46 | 14.4 | 15.5 | 1.2 |
| **TOTAL PAH** | **173** | **231** | **615** | **7831** | **202** | **11555** | **5029** | **293** |

NQ = Not quantified

BDL = Below detection limit
