# Supplementary material for: Polycyclic aromatic hydrocarbons in US and Swedish smokeless tobacco products
Source: Chem Cent J. 2013 Sep 8;7:151. doi: 10.1186/1752-153X-7-151 (PMC3874832; doi:10.1186/1752-153X-7-151)
Supplement: Additional file 5: Table S7 — Pearson coefficients and P-values for correlations between the PAH concentrations (DWB) in the STPs. [file 1752-153X-7-151-S5.docx]

Additional Table S7

Pearson coefficients and P-values for correlations between the PAH concentrations (DWB) in the STPs

|  | NAP | 1-MN | 2-MN | ANY | ANE | FLN | PHEN | ANTH | FLNT | PYR | B[a]A | CHR | B[b]F | B[k]F | B[j]F | B[e]P | B[a]P | PER | I[cd]P | DB[ah]A |
| --- | --- | --- | --- | --- | --- | --- | --- | --- | --- | --- | --- | --- | --- | --- | --- | --- | --- | --- | --- | --- |
| 1-MN | 0.403  0.001 |  |  |  |  |  |  |  |  |  |  |  |  |  |  |  |  |  |  |  |
| 2-MN | 0.347 0.003 | 0.992 0.000 |  |  |  |  |  |  |  |  |  |  |  |  |  |  |  |  |  |  |
| ANY | 0.307  0.010 | 0.776  0.000 | 0.810  0.000 |  |  |  |  |  |  |  |  |  |  |  |  |  |  |  |  |  |
| ANE | 0.351  0.003 | 0.775 0.000 | 0.806  0.000 | 0.981  0.000 |  |  |  |  |  |  |  |  |  |  |  |  |  |  |  |  |
| FLN | 0.326  0.006 | 0.753  0.000 | 0.785  0.000 | 0.982  0.000 | 0.991  0.000 |  |  |  |  |  |  |  |  |  |  |  |  |  |  |  |
| PHEN | 0.290  0.015 | 0.677  0.000 | 0.704  0.000 | 0.919  0.000 | 0.935  0.000 | 0.965  0.000 |  |  |  |  |  |  |  |  |  |  |  |  |  |  |
| ANTH | 0.289  0.015 | 0.681 0.000 | 0.711  0.000 | 0.935  0.000 | 0.946  0.000 | 0.974  0.000 | 0.994 0.000 |  |  |  |  |  |  |  |  |  |  |  |  |  |
| FLNT | 0.268 0.025 | 0.626  0.000 | 0.653  0.000 | 0.878  0.000 | 0.894  0.000 | 0.932  0.000 | 0.992  0.000 | 0.986  0.000 |  |  |  |  |  |  |  |  |  |  |  |  |
| PYR | 0.268  0.025 | 0.625 0.000 | 0.651  0.000 | 0.877 0.000 | 0.890  0.000 | 0.929  0.000 | 0.992  0.000 | 0.985  0.000 | 0.999  0.000 |  |  |  |  |  |  |  |  |  |  |  |
| B[a]A | 0.274  0.022 | 0.633 0.000 | 0.661  0.000 | 0.887  0.000 | 0.896  0.000 | 0.935  0.000 | 0.990  0.000 | 0.989  0.000 | 0.997 0.000 | 0.998  0.000 |  |  |  |  |  |  |  |  |  |  |
| CHR | 0.273  0.022 | 0.624 0.000 | 0.650  0.000 | 0.875 0.000 | 0.884  0.000 | 0.925  0.000 | 0.987  0.000 | 0.983  0.000 | 0.997 0.000 | 0.998  0.000 | 0.999  0.000 |  |  |  |  |  |  |  |  |  |
| B[b]F | 0.279  0.019 | 0.642  0.000 | 0.668  0.000 | 0.889  0.000 | 0.800  0.000 93 | 0.932  0.000 | 0.989  0.000 | 0.981  0.000 | 0.994 0.000 | 0.995  0.000 | 0.995  0.000 | 0.997  0.000 |  |  |  |  |  |  |  |  |
| B[k]F | 0.281  0.018 | 0.643  0.000 | 0.668  0.000 | 0.886  0.000 | 0.889  0.000 | 0.928  0.000 | 0.985 0.000 | 0.976  0.000 | 0.990  0.000 | 0.992  0.000 | 0.991  0.000 | 0.994  0.000 | 0.999  0.000 |  |  |  |  |  |  |  |
| B[j]F | 0.274 0.022 | 0.631 0.000 | 0.656  0.000 | 0.880 0.000 | 0.877  0.000 | 0.918  0.000 | 0.977  0.000 | 0.970  0.000 | 0.985  0.000 | 0.988  0.000 | 0.988  0.000 | 0.992  0.000 | 0.997 0.000 | 0.998  0.000 |  |  |  |  |  |  |
| B[e]P | 0.272 0.023 | 0.624  0.000 | 0.649  0.000 | 0.873  0.000 | 0.874 0.000 | 0.916  0.000 | 0.978 0.000 | 0.970  0.000 | 0.986  0.000 | 0.989 0.000 | 0.988  0.000 | 0.993  0.000 | 0.998  0.000 | 0.998 0.000 | 0.999 0.000 |  |  |  |  |  |
| B[a]P | 0.281 0.018 | 0.649  0.000 | 0.677  0.000 | 0.904 0.000 | 0.903  0.000 | 0.941  0.000 | 0.987  0.000 | 0.985  0.000 | 0.989  0.000 | 0.991  0.000 | 0.995 0.000 | 0.995  0.000 | 0.997  0.000 | 0.996 0.000 | 0.996  0.000 | 0.995  0.000 |  |  |  |  |
| PER | 0.270 0.024 | 0.635 0.000 | 0.662  0.000 | 0.886  0.000 | 0.884  0.000 | 0.925 0.000 | 0.978  0.000 | 0.973  0.000 | 0.983  0.000 | 0.985  0.000 | 0.987 0.000 | 0.990 0.000 | 0.996 0.000 | 0.996  0.000 | 0.997  0.000 | 0.997  0.000 | 0.995  0.000 |  |  |  |
| I[cd]P | 0.307 0.010 | 0.645 0.000 | 0.671  0.000 | 0.893  0.000 | 0.888 0.000 | 0.925  0.000 | 0.958  0.000 | 0.953  0.000 | 0.955  0.000 | 0.959  0.000 | 0.957  0.000 | 0.961  0.000 | 0.971  0.000 | 0.972  0.000 | 0.975  0.000 | 0.973  0.000 | 0.973  0.000 | 0.970  0.000 |  |  |
| DB[ah]A | 0.305  0.010 | 0.639 0.000 | 0.666  0.000 | 0.887 0.000 | 0.887 0.000 | 0.925  0.000 | 0.971 0.000 | 0.962  0.000 | 0.971  0.000 | 0.974  0.000 | 0.973  0.000 | 0.974  0.000 | 0.982 0.000 | 0.983  0.000 | 0.982  0.000 | 0.981  0.000 | 0.981  0.000 | 0.980  0.000 | 0.976  0.000 |  |
| B[ghi]P | 0.313 0.008 | 0.620 0.000 | 0.643  0.000 | 0.865  0.000 | 0.858 0.000 | 0.898  0.000 | 0.936  0.000 | 0.928  0.000 | 0.937  0.000 | 0.941  0.000 | 0.935  0.000 | 0.942  0.000 | 0.952  0.000 | 0.954  0.000 | 0.958  0.000 | 0.958  0.000 | 0.953  0.000 | 0.950  0.000 | 0.994  0.000 | 0.957  0.000 |

Pearson coefficients are shown in the upper part of cell and p-values in the lower part
